# Supplementary material for: Complex‐centric proteome profiling by SEC‐SWATH‐MS
Source: Mol Syst Biol. 2019 Jan 14;15(1):e8438. doi: 10.15252/msb.20188438 (PMC6346213; doi:10.15252/msb.20188438)
Supplement: Supplementary file 8 — Dataset EV7 [file MSB-15-e8438-s008.zip › feature_plots_string/Q9UBL3.pdf]

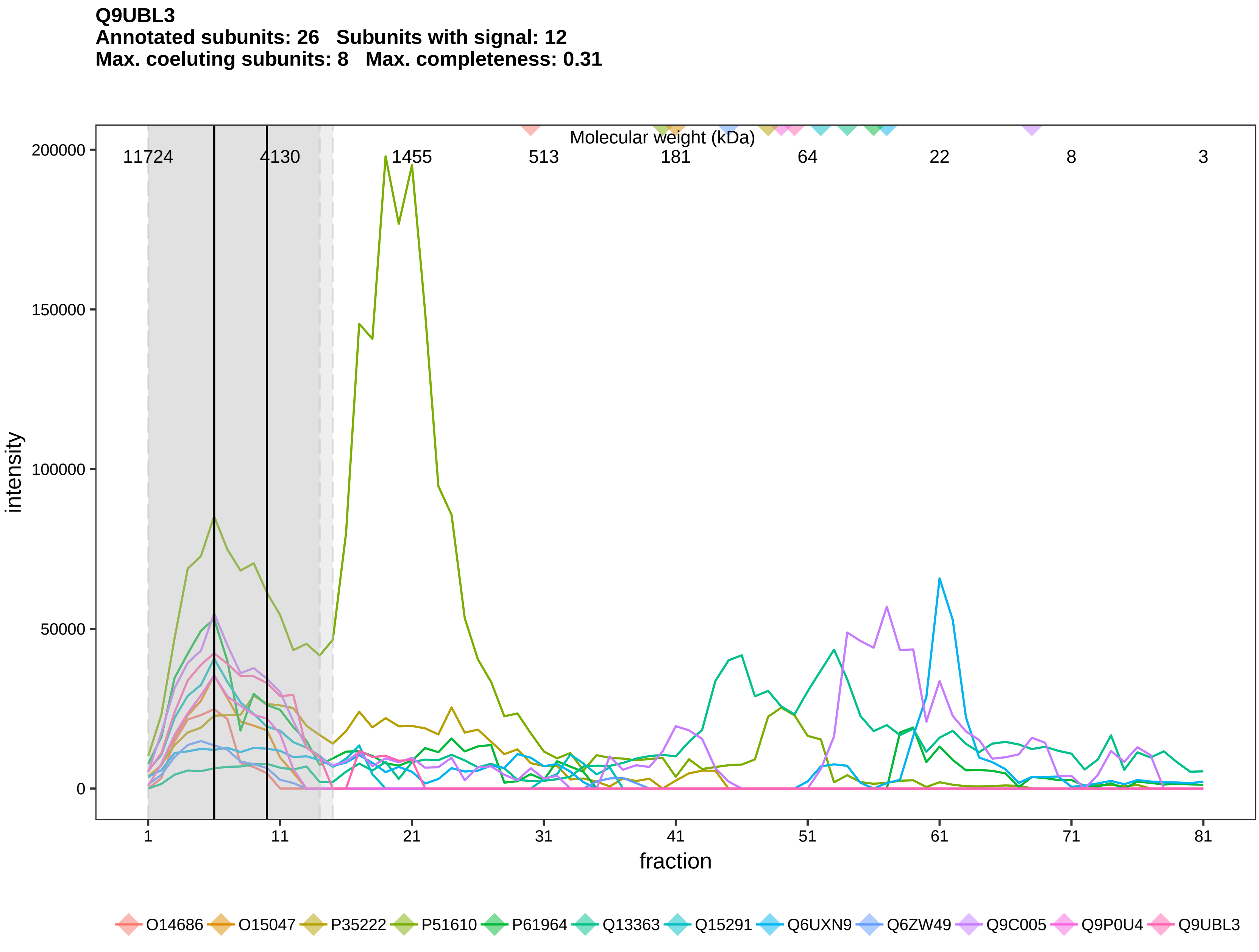
Q9UBL3
Annotated subunits: 26   Subunits with signal: 12
Max. coeluting subunits: 8   Max. completeness: 0.31
Molecular weight (kDa)
11724
4130
1455
513
181
64
22
8
3
200000
150000
100000
50000
0
intensity
1
11
21
31
41
51
61
71
81
fraction
O14686
O15047
P35222
P51610
P61964
Q13363
Q15291
Q6UXN9
Q6ZW49
Q9C005
Q9P0U4
Q9UBL3
